# The effect of novel mosquito bite prevention tools on *Anopheles minimus* landing and key secondary endpoints: semi-field evaluations in Thailand

**DOI:** 10.1186/s12936-024-05188-3

**Published:** 2024-12-18

**Authors:** Élodie A. Vajda, Amanda Ross, Manop Saeung, Arissara Pongsiri, David J. McIver, Allison Tatarsky, Nakul Chitnis, Jeffrey Hii, Jason H. Richardson, Michael Macdonald, Sarah J. Moore, Neil F. Lobo, Theeraphap Chareonviriyaphap, Alongkot Ponlawat

**Affiliations:** 1 University of California, San Francisco, 550 16th Street, San Francisco, CA 94158 USA; 2 Swiss Tropical and Public Health Institute, Kreuzstrasse, 2CH-4123 Allschwil, Switzerland; 3 University of Basel, Petersplatz 1, CH-2003 Basel, Switzerland; 4 Ifakara Health Insititute, PO Box 74, Bagamoyo, Tanzania; 5 University of Notre Dame, Notre Dame, St. Joseph County, IN 46556 USA; 6 Kasetsart University, 50 Thanon Ngamwongwan, Lat Yao, Chatuchak, Bangkok, 10900 Thailand; 7 Armed Forces Research Institute of Medical Sciences, 315/6 Ratchawithi Road, Thung Phaya Thai, Ratchathewi, Bangkok, 10400 Thailand; 8 Liverpool School of Tropical Medicine, Innovative Vector Control Consortium, Pembroke Place, Liverpool, L3 5QA UK

## Abstract

**Background:**

The Greater Mekong Subregion (GMS) aims to eliminate all human malaria by 2030 and is making substantial progress toward this goal, with malaria increasingly confined to forest foci. These transmission foci are predominantly inhabited by ethnic minorities, local populations, and rural mobile and migrant populations working in mining and agriculture. The recommendations of the World Health Organization (WHO) on malaria elimination states that small population groups which constitute a large proportion of the malaria transmission reservoir should benefit from targeted strategies to reduce transmission overall. These population groups are exposed to malaria vector bites during the day due to *Anopheles* daytime biting, and during the night, due to low bed net use and open sleeping structures. Such characteristics limit the effectiveness of the WHO core vector control strategies [indoor residual spraying (IRS), insecticide-treated nets (ITNs)], which target indoor resting and indoor feeding mosquitoes. Interventions that target daytime and outdoor resting or biting mosquitoes, and which complement IRS and ITNs and drug strategies, may hasten a decline in the malaria burden.

**Methods:**

This study evaluated two transfluthrin- and one metofluthrin-based volatile pyrethroid spatial repellents (VPSRs), and etofenprox insecticide-treated clothing (ITC) with and without a topical repellent in a semi-field system (SFS) at two research sites in Thailand, across two trial rounds. The study estimated the protective efficacies of the vector control tools against two pyrethroid-susceptible *Anopheles minimus* strains in the form of 15 interventions, including a combined VPSR and ITC intervention. The interventions’ modes of action were studied by measuring their impact on mosquito landing, and on key life history traits known to affect vectoral capacity (knockdown, post-exposure blood feeding, and 24-h mortality) using a block-randomized crossover design. The odds ratio (OR) for each intervention compared to the control on each outcome was estimated.

**Results:**

All interventions substantially reduced *An. minimus* landings and prevented more than 50% mosquito landings when new (VPSRs) or unwashed (treated clothing). In addition to landing reduction, all interventions decreased post-exposure blood feeding, induced knockdown and increased mortality at 24 h. The VPSR interventions were generally more protective against landing than the treated clothing intervention. The combined intervention (VPSR + ITC) provided the greatest protection overall.

**Conclusion:**

This SFS evaluation indicates an effect of these VPSR and ITC interventions in reducing *An. minimus* landing for the user, and indicates their potential for community protection by secondary modes of action. This study demonstrates the utility of SFS trials in the evaluation of bite prevention tools and emphasizes the need for multiple evaluations at different sites. It also highlights possible sources of biases observed, including the measuring of mosquito landing rather than biting, weather parameters, and low mosquito recapture.

**Supplementary Information:**

The online version contains supplementary material available at 10.1186/s12936-024-05188-3.

## Background

Over the last decade, amplified malaria control has successfully reduced the Greater Mekong Subregion’s (GMS) malaria burden. From 2000 to 2020, the GMS recorded a 56% decrease in malaria, and an 89% reduction in *Plasmodium falciparum* cases [1]. Today, malaria transmission in the region is confined to pockets of transmission along international borders, in forests, and along forest fringes [2]. These residual malaria transmission foci are predominantly inhabited by ethnic minorities, local populations that live in and around forests, and rural mobile and migrant populations working in mining and in agriculture [2–4].

Gaps in protection are places and times in which people are exposed to potentially infectious mosquito bites. In the context of forest malaria, forest-goers and -dwellers are exposed to vector bites during the day due to *Anopheles* exhibiting daytime biting (e.g., *Anopheles dirus*, *Anopheles maculatus*), and during the night, due to low bed net use [3, 4]. Prolonged visits to the forest for economic activities, during which individuals sleep in partially or completely open sleeping structures, as well as nocturnal work (for example, rubber tapping), are two of the main malaria risk factors in the forest [5]. These living circumstances limit the effectiveness of traditional homestead-centric vector control interventions [indoor residual spraying (IRS), insecticide-treated nets (ITNs)] [5], which target indoor and late-night feeding mosquitoes [3, 6]. Thus, for the GMS to attain its ambitious malaria elimination goals, new approaches to vector control are needed to target these forest-based gaps in protection.

There are many promising novel vector control tools in the development pipeline; for example, volatile pyrethroid spatial repellents (VPSRs) [7–9], insecticide-treated clothing (ITC) [10–12], genetically modified mosquitoes [13], attractive targeted sugar baits (ATSBs) [14], and endectocides (e.g., ivermectin) [15]. This study focuses on two types of interventions with overlapping modes of actions: VPSRs and ITC (ITC was sometimes paired with a topical repellent) (Table 2). VPSRs use pyrethroids that act in the vapor phase and work by preventing human-vector contact primarily through *non-contact* irritancy, non-contact excitorepellency (the combined effects of both irritation from coming into direct contact with a treated area and the tendency to avoid treated areas due to their repellent properties), spatial repellency, landing inhibition, feeding inhibition, and sublethal incapacitation [16, 17]. On the other hand, ITC treated with pyrethroids primarily protects humans from mosquito bites through *contact* irritancy, contact excitorepellency, short-range *non-contact* excitorepellency, and feeding inhibition [10–12]. Finally, synthetic topical repellents such as picaridin and DEET, provide personal protection against mosquito bites via short-range actions such as olfactory attractor inhibition [18, 19], non-contact irritancy, and/or contact irritancy [20, 21]. Combining ITC with a topical repellent could enhance bite protection [22]. Thus, VPSRs, ITC, and topical repellents might be appropriate for use amongst forest-exposed populations, due to their ability to provide protection against vector biting outside the peridomestic area [23].

To date, the WHO has not issued an official stance on the utilization of VPSRs, ITC, and topical repellents in public health vector control. While the WHO does not formally endorse the applications of ITC and topical repellents, it suggests these interventions for personal protection and considers their use for high-risk groups who may not benefit from other vector control measures [24]. The 2017 Global Vector Control Response Framework by WHO/UNICEF emphasizes the importance of research and development for novel tools, including VPSRs [25].

Recent research, using a stochastic transmission model based on controlled experiments with transfluthrin-treated material VPSRs applied under the roof of houses, not only demonstrated a reduction in vector landing (personal protection) but also highlighted their ability to kill vectors and reduce blood feeding, resulting in substantial decreases in vectoral capacity [26]. This suggests that VPSRs have potential for community-wide protection in addition to personal protection. Similar findings have been observed from a number of semi-field system (SFS) experiments [7–9, 27–29] and field studies of VPSRs [30], including recent interim analysis results from the Unitaid Advancing Evidence for the Global Implementation of Spatial Repellents (AEGIS) Kenya trial [31]. Despite this growing evidence, a recent landscape analysis and review on repellents for mosquito control highlighted the continued need for more evidence of epidemiological impact and a better understanding of the interventions' modes of action [32]. Clarifying the modes of action of VPSRs and ITC is crucial for understanding observed epidemiological impacts in the field [26].

Therefore, this study aimed to describe the modes of action by which two transfluthrin-based VPSRs, one active emanator (device requires activation or user interaction to release the repellent) and one passive emanator (device releases insect-repelling chemicals over time, providing continuous protection against mosquito bites), one passive metofluthrin-based VPSR, and etofenprox-treated clothing (sometimes paired with a picaridin topical repellent) operate in the SFS against *An. minimus* (an important malaria vector in Southeast Asia [29, 30]). Transfluthrin is a synthetic pyrethroid that has been demonstrated to induce knockdown (KD), mortality, and blood feeding inhibition, in addition to repellency against *Anopheles*, including *Anopheles* strains resistant to pyrethroids [8, 9, 17, 28, 29, 33–35]. Metofluthrin is also a synthetic pyrethroid that has been shown to induce KD and mortality in addition to repellency [27, 36, 37]. Etofenprox is a synthetic pyrethroid-like ether insecticide used to treat clothing to protect the user from mosquito bites. The structure of etofenprox renders it more stable, less toxic to humans, but more toxic to mosquitoes than permethrin formulations currently used on ITC [38, 39]. Etofenprox functions by attacking the neuronal axon of the mosquito, protecting humans from mosquito bites primarily through contact irritancy [39].

This study was conducted in the SFS at two research sites in Thailand [Armed Forces Research Institute of Medical Sciences (AFRIMS) and Kasetsart University (KU)]. The VPSR and ITC products were simultaneously tested in the two sites in the form of 15 interventions distributed across two test rounds, for their respective protective efficacy against two pyrethroid-susceptible *An. minimus* strains. The impact of the interventions was estimated based on mosquito landing inhibition (primary outcome). As the study was conducted in a closed system, it was also possible to evaluate immediate KD, post-exposure blood feeding inhibition, and mortality at 24 h (secondary outcomes). These four outcomes represent substantial intervention effects that impact disease transmission through modes of action including repellence, diversion, disarming, feeding inhibition, and mortality (outcomes and their associated modes of action are defined in Table 2).

This SFS evaluation is part of a broader, multi-staged, research program called ‘Project BITE’ (Bite Interruption Towards Elimination) (2020–2023). Project BITE used a mixed-methods, phased approach to evaluate personal mosquito bite prevention interventions towards informing their use for protecting mobile and forest human populations in Southeast Asia against malaria. As the first stage of Project BITE, this SFS study generated data on the modes of actions of these VPSR and ITC interventions towards better understanding their potential for public health impact and served as a precursor to the field evaluation in Cambodia of a subset of these interventions [40]. These SFS data were also fitted to a stochastic model using a Bayesian inference approach to predict intervention impacts on transmission of *P. falciparum* malaria by *An. minimus* [41].

## Methods

### Study sites and semi-field screen house

This study was replicated at two research sites in Thailand: AFRIMS and KU. The experiments occurred in the SFS for each site, in Kamphaeng Phet (AFRIMS) and in Kanchanaburi (KU) provinces. An SFS consists of a large, screened cage (AFRIMS: V = 806.4 m^3^; KU: V = 560.0 m^3^) that allows controlled experiments with disease-free, laboratory-reared mosquitoes of known insecticide susceptibility status and physiological age to be conducted under ambient climatic conditions [23].

### Experimental chambers

**Fig. 1:**
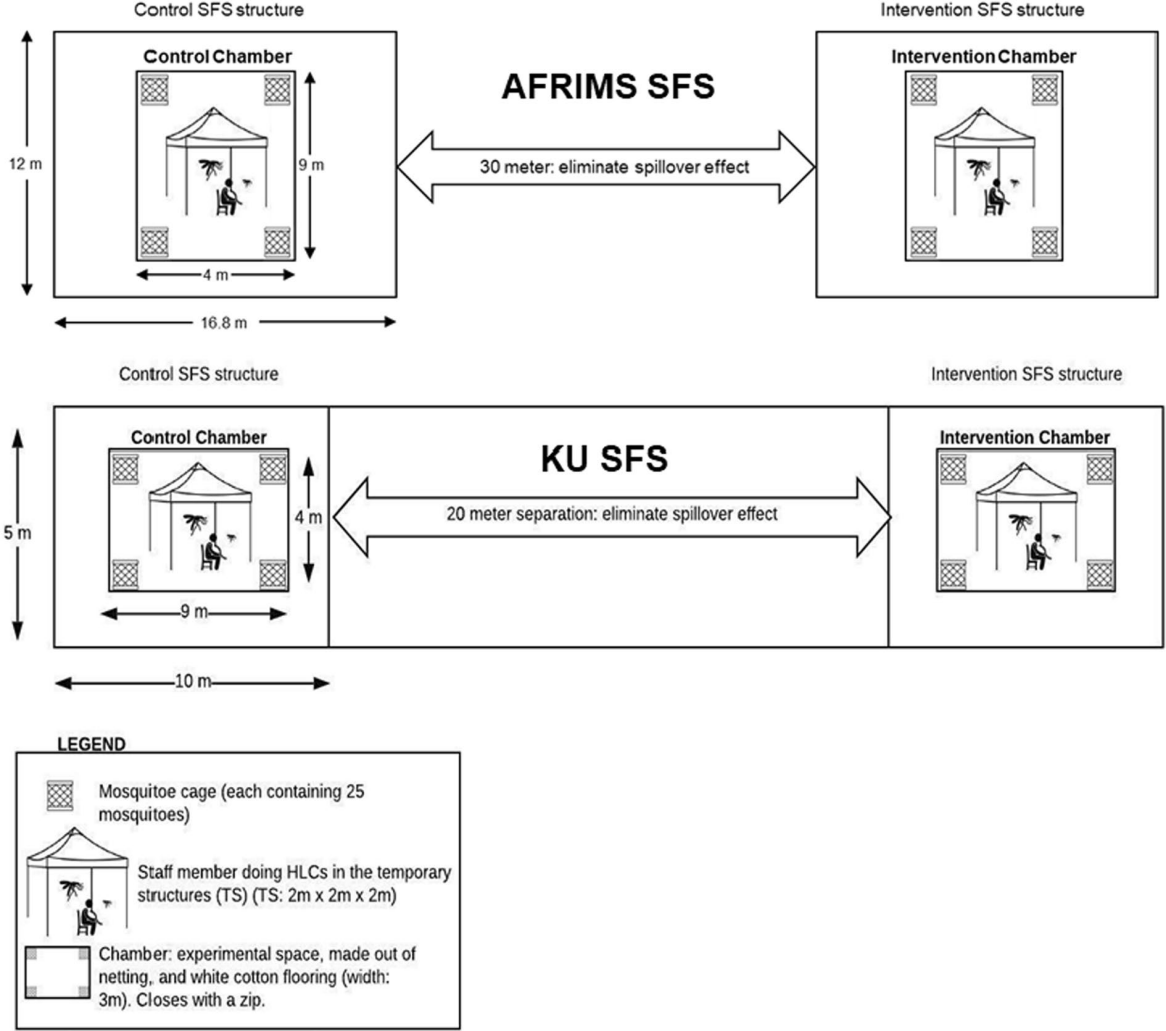
Experimental set-up in the SFS enclosures at AFRIMS (top) and KU (bottom)

**Fig. 2:**
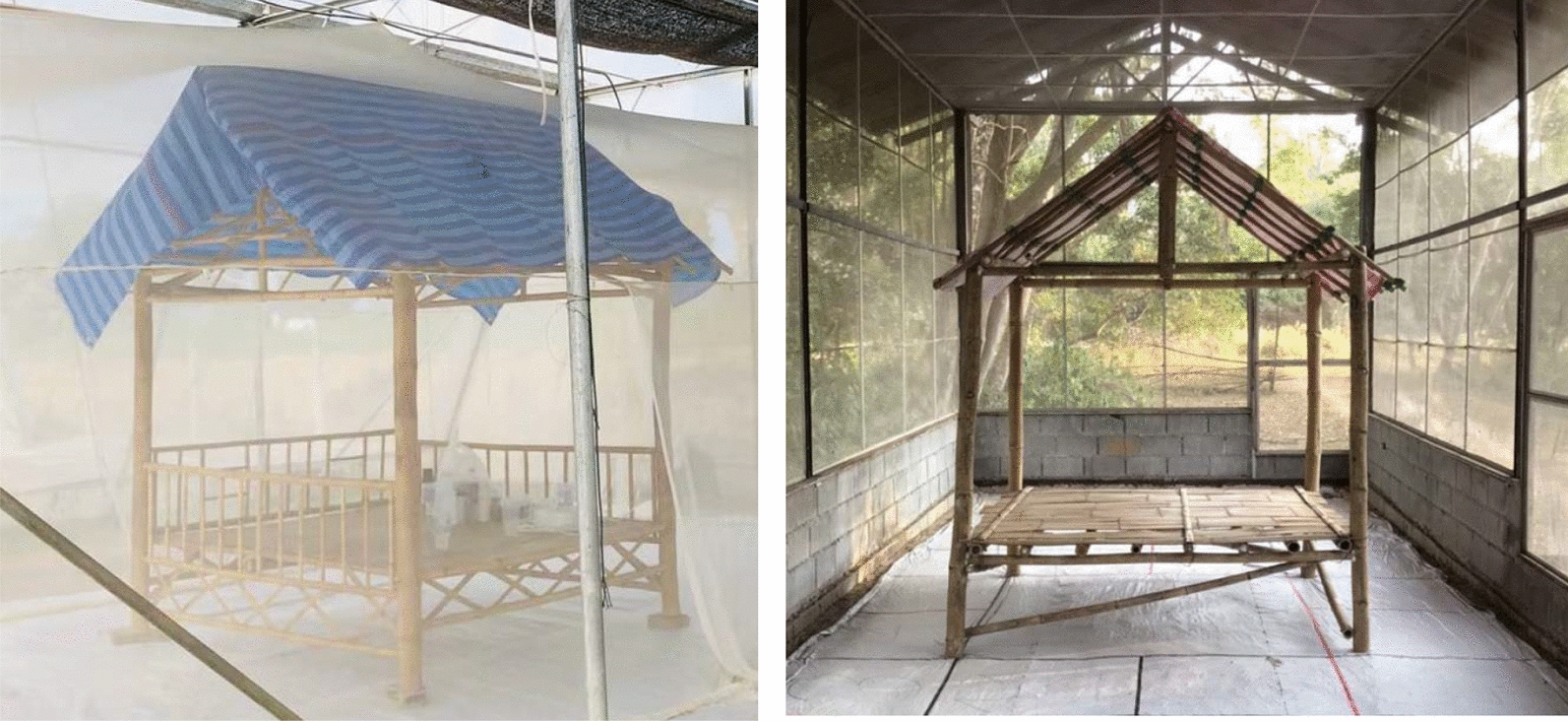
Bamboo temporary open structure at AFRIMS (left) and at KU (right)

At each site, the evaluation was conducted in two identical experimental chambers made of untreated bed net material and of a white cloth (cotton) floor, each measuring 9 × 4 × 3 m (V = 108.0 m^3^) with 20 m between the two chambers (Fig. 1). The white cloth inner chamber aids mosquito recapture for measurement of secondary effects. Prior to the onset of the trials, spillover testing conducted at both AFRIMS and KU found no spillover effects between chambers, thus ensuring independence of observations, even when using volatile pyrethroids. A temporary open structure representative of open sleeping structures (2 × 2 × 2 m) (V = 8.0 m^3^) as observed in the forest in Cambodia was constructed inside each experimental chamber from four bamboo poles (2 m) overlaid with a tarpaulin placed/attached to the top with an overhang of 30 cm (Fig. 2).

### Study design

This study applied a block-randomized crossover design. The intervention and a control were randomly assigned to one of two separate chambers within the SFS for a block of 4 days. Blocks of 4 days were chosen: (1) to emulate the possible real-world scenario of accumulation of active ingredient, (2) blocks of one day would increase the length of the study considerably, due to the wash out days in between, (3) prior studies using this method have yielded reliable results [28, 42], and (4) to decrease the demand on staff carrying out the study. Wash out refers to the period of time in which a space is left empty, with no intervention present and no activity taking place, in order to rid the space of any residual active ingredient from the intervention. This is to ensure that the effects of one intervention or trial do not impact the results of any other evaluated afterward. Successful wash out of active ingredients was verified by running a cone bioassay on the walls of the chamber using a pyrethroid susceptible mosquito strain. The open structure’s tarp roof was also aired out and underwent a cone bioassay. If no vector knock down was observed, then the compartment was designated as clean.

In each block of four days (i.e., four replicates), four staff members rotated between compartments daily to control for any biases caused by individual attractiveness to mosquitoes or ability to capture mosquitoes which may impact upon intervention efficacy. After each experiment (two blocks of four days, i.e., eight replicates), each staff member had evaluated each treatment twice in each chamber. After each block of 4 days, the intervention was switched between chambers, to control for any possible bias between the two chambers due to differential air flow, following a wash out period of two days for the spatial repellent arms.

### Mosquitoes

Two susceptible *An. minimu*s strains were used for these trials: AFRIMS’s lab strain, and KU’s CDC lab strain. AFRIMS’ *An. minimus* laboratory strain has been reared in the Insectary Section of the Department of Entomology at AFRIMS for over two decades. KU’s *An. minimus* CDC laboratory strain was originally obtained from the Bureau of Vector-borne Diseases, Department of Disease Control, Ministry of Public Health, Thailand, and has been reared at KU’s insectary since 1993 [43]. At both research sites, mosquito colonies were reared under conditions of 25 ± 2 °C and 80 ± 10% humidity. Colony susceptibility to insecticides was confirmed before the study started using WHO tube tests [44, 45]. Insecticides tested include deltamethrin, etofenprox, permethrin, and transfluthrin. Transfluthrin and etofenprox are active ingredients of the interventions under study, while permethrin and deltamethrin are present in insecticide treated nets found in the field study area. Insecticide resistance testing of both AFRIMS’ and KU’s *An. minimus* strains were conducted at KU. All mosquitoes used for SFS tests were five to eight days old, nulliparous females, and sugar-starved for eight hours to ensure avidity to blood feeding. Selection of hungry female mosquitoes was confirmed by placing a hand close to the side and aspirating those mosquitoes which were responsive to the hand.

To allow for acclimatization, mosquitoes were transferred from the insectary to a holding chamber thirty minutes before the experiment was initiated. The holding chamber was separated from the experimental chamber, where the intervention being evaluated would be present, in order to prevent any impact of the intervention on the mosquitoes before the trial began.

### Applications of interventions

Fifteen interventions were derived from four test products, across two test rounds from 2020 to 2021 (referred to as R1 and R2) (Table 1). Note that 20% picaridin topical repellent (SC Johnson) was also used in combination with four interventions, but was never tested for efficacy on its own. The picaridin repellent was selected for this study as it has previously been demonstrated to be safe and effective against Southeast Asian vectors of malaria [46].

**Table 1:** Interventions tested in the SFS

Intervention (test round)	Intervention description	Control
1.BiteBarrier (new) (BB-new) (R1, R2)	Passive transfluthrin-based VPSR, previously unused and unopened. Two BiteBarrier units were hung on opposing sides of the intervention temporary open structure, two hours prior to onset of collections. The same BiteBarrer units were used for the eight replicates Additional product information: BiteBarrier, formerly known as PIRK, PIC Corp, is composed of two 25 × 25 cm sheets (4 sheets total), and is intended for hanging inside rooms. BiteBarrier is transfluthrin-based (98.68%), an odorless active ingredient manufactured by *Envu*, known for its excellent safety profile in mammals [73]. Depending on their exposure levels to this active ingredient, transfluthrin can effectively prevent mosquito biting and induce mosquito mortality [17, 74]. 11/21/2024 11:36:00 AM	No devices hung. Collectors wore short sleeves and short trousers, with a net jacket and covered shoes. Both legs (knee to ankle) exposed
2.BiteBarrier (aged 20 days) **(**BB-20) (R1)	BiteBarrier was ‘aged’ for twenty days. This process involved hanging the BiteBarrier sheets on the morning of ‘ageing’ day one using hangers and clips in a shaded, outdoor structure located away from human circulation and protected from wind, temperature fluctuations (acceptable temperature range 23.9–32.3 ºC), and sun exposure. The BiteBarrier units were left in the same location for twenty days. Each BiteBarrier were separated from one another by at least one meter. On the morning of day 21, the BiteBarrier units were removed and individually carefully wrapped each in aluminum foil and stored in the refrigerator at 4 ºC. Aged BiteBarriers had to be used within the following two to three days to be considered as ‘aged 20 days’). The same aged BiteBarriers were used for the eight replicates	
3.BiteBarrier (aged 30 days) (BB-30) (R2)	BiteBarrier was ‘aged’ for thirty days by being hung in the same location at both institutes using the same ageing method as for BB-20, and were retrieved at day 31 for trial use. The same aged BiteBarriers were used for the eight replicates	
4.Fuyi Sin Olor (Fuyi) (R1)	Active transfluthrin-based VPSR, in the form of a hand-held, aerosol with pulsation spray can. To apply in the temporary open structure, the applier stood in the middle of the open structure, and sprayed once each of the four corners of the structure towards the eaves, over 0.5 m (~ 1.64 feet) away from the substrate. Application of Fuyi Sin Olor in the open structure was done once only per night, five minutes before onset of the HLCs Additional product information: Fuyi Sin Olor is composed of 3.05% transfluthrin and is manufactured by SC Johnson & Son, Inc, Racine, WI, USA	
5.SumiOne (new) (R2)	Passive metofluthrin-based VPSR, previously unused and unopened. Two SumiOne units were hung on opposing eaves of the intervention temporary open structure, two hours prior to onset of collections. The same SumiOne units were used for the eight replicates Additional product information: The SumiOne passive emanator device is comprised of a methacrylate polymer net impregnated with 10% w/w (ca. 0.217 g) of the synthetic, volatile pyrethroid metofluthrin (manufactured by Sumitomo Chemical Company Ltd., Tokyo, Japan). The formulation is currently registered in various formulations in Australia, Singapore, Malaysia, and Thailand. The device’s impregnated net is contained within a 95 × 160 mm plastic holder, intended for hanging inside rooms, against a wall. Metofluthrin is characterized by its elevated volatility, low mammalian toxicity, and important lethal/KD effects on mosquitoes [75]	
6.Etofenprox-treated ranger uniform (0 washes) (EtoR-0) (R1)	Long-sleeved and round collar shirt and long trousers made of 50% cotton and 50% nylon textile, worn with a net jacket. Uniforms were treated by hand, using the etofenprox spray application bottles (700 mL spray bottles (7.0% etofenprox) with a calibrated nozzle. The etofenprox was applied according to manufacturer’s instructions: the bottle was held 15–20 cm away from the garment to allow spraying on fabric for a treatment level of 2.0 g/m^2^. Using slow, sweeping motion, the garments were evenly coated for approximately 30 s on each side. Garments were air dried for 2 h by being hung outside. Unwashed Additional product information: Etofenprox (Perimeter ETO Insect Guard formulation) is an insecticide manufactured by *Mitsui Chemicals* and formulated exclusively by *Pine Belt Processing*, a wholly owned subsidiary of *Warmkraft, Inc*. Etofenprox is approved by the United States Environmental Protection Agency (US EPA) specifically designed for treating clothing worn by the US military [76]	Untreated uniforms with boot footwear, with a net jacket. Trousers were not rolled up to the knees in order to preserve the functionality of the ranger uniform
7.Etofenprox-treated ranger uniform (20 washes) (EtoR-20) (R1)	Same intervention as for EtoR-0, except washed 20 times. To wash the clothing twenty times, washing and drying were repeated once per day, every day, for 20 days. Clothing was washed by washing machine, on the cold setting, using soap flakes. Washed clothing was air dried by being laid out flat on tarps in the shade	
8. Etofenprox-treated civilian clothing (short-sleeved shirts and short trousers) (0 washes) (Eto-Sh-0) (R1)	Short-sleeved t-shirt and short trousers (knee-length), worn with a net jacket. Clothing made of 100% cotton. Same etofenprox treatment method as for EtoR-0 and EtoR-20	Matched civilian clothing (100% cotton), short-sleeved t-shirt and short trousers, with a net jacket and closed shoes
9.Etofenprox-treated civilian clothing (short-sleeved shirts and short trousers) (20 washes) (Eto-Sh-20) (R1)	Same intervention as Eto-Sh-0, except washed 20 times. The same washing method as for EtoR-20 was applied	
10.Etofenprox-treated ranger uniform with 20% picaridin (0 washes) (EtoR-0-Pi) (R1)	Same uniform and etofenprox treatment as for EtoR-0 and EtoR-20. A 20% picaridin (191 g/L) topical repellent (OFF! Tropical Strength Insect Repellent Spray, SC Johnson & Son Pty Ltd) was applied 1 ml/600 cm^2^ so total ml to exposed hands of the collector conducting the HLCs and wearing the treated clothing. Prior to each collection shift, the collector applied the topical repellent evenly to the hands, as these were the only areas of the body not covered by the treated uniform	Untreated uniforms with boot footwear, along with net jacket. Trousers were not rolled up to the knees in order to preserve the functionality of the ranger uniform
11.Etofenprox-treated ranger uniform with 20% picaridin (20 washes) (EtoR-20-Pi) (R1)	Same intervention as for EtoR-0-Pi, except uniform washed 20 times (same wash methods as for EtoR-20 and EtoC-20)	
12.Etofenprox-treated civilian clothing (short-sleeved shirts and short trousers (knee-length)) with 20% picaridin (0 washes) (Eto-Sh0-Pi) (R1)	Same civilian clothing and etofenprox treatment as for Eto-Sh-0 and Eto-Sh-20. 20% Picaridin topical repellent was applied at 1 ml/600 cm^2^ to all exposed skin of the collector conducting the HLCs and wearing the treated clothing. Prior to each collection shift, the collector applied the topical repellent evenly to the areas of the body not covered by the treated clothing or net jacket	Matched civilian clothing (100% cotton), short-sleeved t-shirt and short trousers, with a net jacket and closed shoes
13.Etofenprox-treated civilian clothing (short-sleeved shirts and short trousers (knee-length)) with 20% picaridin (20 washes) (Eto-Sh20-Pi) (R1)	Same intervention as for Eto-Sh0-Pi, except civilian clothing washed 20 times (same wash methods as for EtoC-20 and EtoR-20)	
14.Etofenprox-treated civilian clothing (short-sleeved shirts and long trousers) with 20% picaridin (0 washes) (EtoCL-0) (R2)	Same intervention as for Eto-Sh0-Pi, except collectors wore long trousers instead of short trousers	Matched civilian clothing (100% cotton), short-sleeved t-shirt and long trousers, with a net jacket and closed shoes
15. Combined interventions (BBEtoPI**)** **(**R2**)**	Two BiteBarrier units were hung on opposing eaves of the intervention temporary open structure, two hours prior to onset of collections. The same BiteBarrier units were used for the eight replicates. At the same time, the collector also applied intervention EtoCL-0	

### Experimental procedure

The experiment started when mosquitoes were released remotely by gently pulling strings connected to the releasing cages, allowing mosquitoes to approach staff in all directions to mimic the natural environment. Each replicate consisted of six-hour collections per night (20h00–02h00), with continuous incandescent lighting coming from the center of the bamboo temporary open structures. A single release of 100 mosquitoes was done at 20h00 in each experiment to coincide with mosquitoes’ peak circadian activity. Mosquitoes were collected via human landing catches (HLCs) by a single collector using the given intervention according to label instructions (Table 2). Environmental conditions (temperature, relative humidity, air flow) of the test environment were monitored and recorded hourly from 20h00 to 02h00 using an HOBO data logger (Onset Computer Solutions MA, USA).

**Table 2:** Primary and secondary outcomes measured for each product tested in the SFS

Outcome	Definition	Associated modes of action
*Landing inhibition*	Proportion of mosquitoes landing	Protection to user: landing inhibition leads to ‘repellence**’** (the mosquito moves away from an otherwise attractive host). This is personal protection [26]
*Immediate Knockdown (KD)*	Proportion of mosquitoes incapacitated (unable to stand or fly in a coordinated manner)	‘Disarming’ of mosquitoes, the temporary incapacitation of the mosquito for one or more days before re-entering the feeding cycle, provides protection to the intervention users and non-users nearby, thus preventing ‘diversion’ to non-users and providing community protection [26]
Post-exposure blood feeding inhibition*	Out of mosquitoes that were offered a post-exposure blood meal, the number of mosquitoes that immediately blood fed	‘Feeding inhibition’, the disruption of blood feeding, provides protection to the intervention users, and triggers ‘disarming’ of mosquitoes which in turn prevents ‘diversion’ to non-users and reduces mosquito lifetime reproductive output that will protect intervention users and non-users from malaria; this is community protection [41]
*Mortality at 24 h*	Proportion of dead *An. minimus* mosquitoes captured per treatment	Reduction in mosquito survival (‘death’) will protect intervention users and non-users from malaria; this is community protection [26]

^*^At AFRIMS, blood feeding was not measured for BB-20. At KU, blood feeding was not measured for BB-20, Eto-Sh-20, EtoR-20, and EtoR-20-Pi

### Ethical statement

At KU, ethics approval for the study was given by the Research Ethics Review Committee for Research Involving Human Research Participants, Kasetsart University (No. CAO63/035). At AFRIMS, the study was carried out by trained entomologists from the Department of Entomology, USAMD-AFRIMS, following approved WRAIR Institutional Review Board (IRB)/HSPB protocols (WRAIR#2709).

### Primary and secondary outcome measurements

#### Primary outcome: landing inhibition

**Fig. 3:**
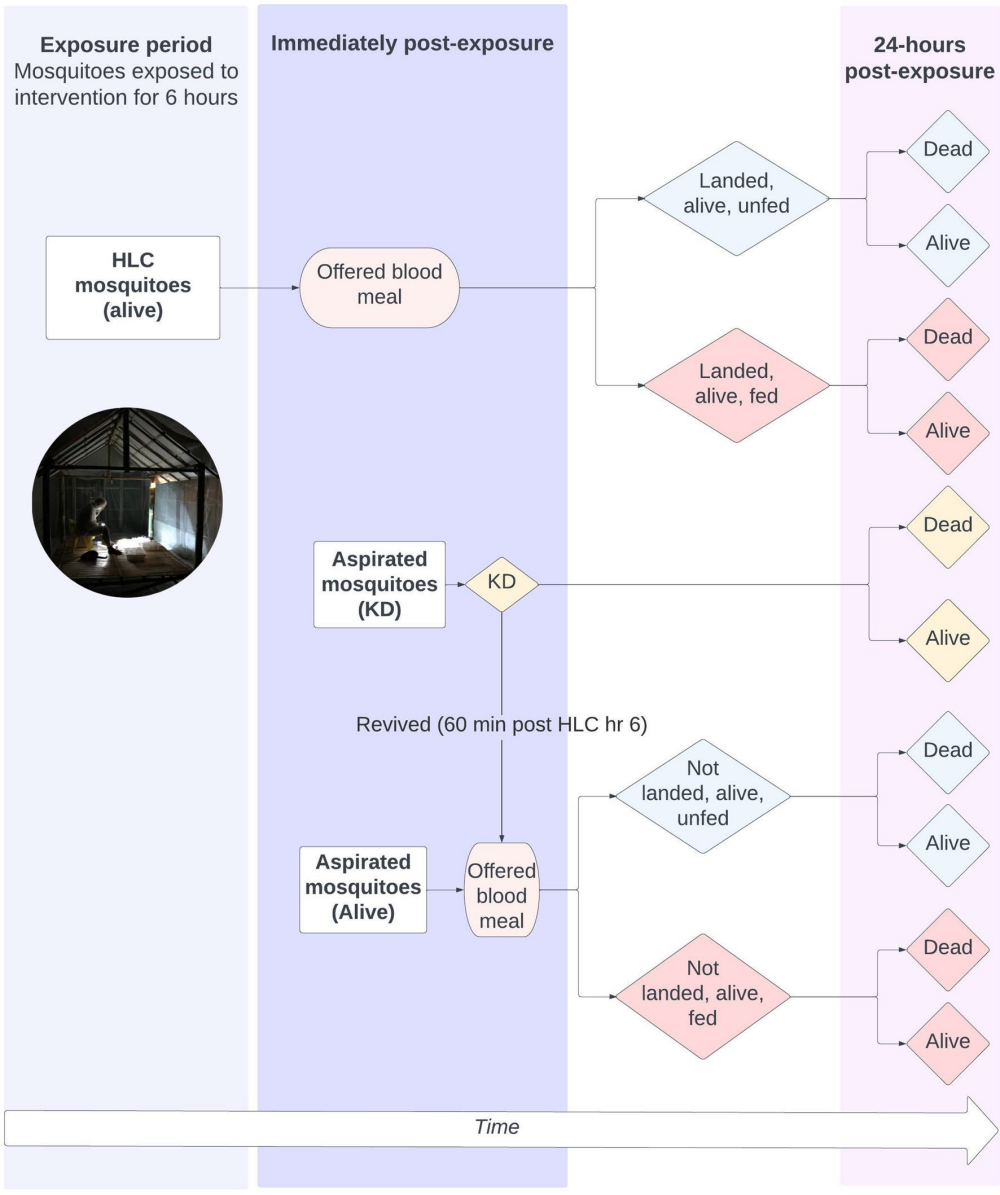
Workflow of measured outcomes

Mosquito landing using HLCs were measured as a proxy for mosquito biting. HLCs were conducted for 45 min per each of the six collection hours in each replicate, with each HLC collection cup labeled for 15-min intervals. At the end of each 15-min interval, used collection cups were placed in a plastic box to reduce exposure of collected mosquitoes to the active ingredient before transfer to the insectary. (Table 2, Fig. 3).

#### Secondary outcomes: immediate knock down, blood feeding inhibition, and mortality at 24-h

At the end of each six-hour replicate, remaining mosquitoes were collected using a CDC backpack aspirator from the floor, around the experimental chamber, and from underneath the roof of the temporary open structure, in both the intervention and control chambers. Backpack aspirator collections were carefully conducted, with two-minute collection periods per collection cup, to reduce handling related mortality. After completion of aspirations, mosquitoes were returned to the insectary to be scored as either alive or KD*.* Mosquitoes were considered KD if they could not stand, fly in a coordinated manner, or if they lay on their backs moving legs and wings but unable to take off, or, if able to take off, fell down immediately. During KD scoring for up to 60 min post HLC hour 6, should any KD mosquito revive and appear alive, this mosquito was considered as alive. Immediately prior to being offered a blood meal, mosquitoes at 15-min HLC intervals were combined into a single cup for each HLC hour. Next, all live mosquitoes were offered a blood meal, as soon as logistically possible, following the completion of the exposure period, while noting the period between final exposure and blood feeding. Finally, following post-exposure blood feeding, live mosquitoes were held under insectary conditions with access to a 10% sugar meal and observed for mortality at 24 h. At the 24-h scoring time, mosquitoes were scored as blood fed alive, blood fed dead, unfed alive, or unfed dead (Table 2, Fig. 3).

### Sample size calculation

The sample size was based on the power to detect a true difference between the intervention and control groups as significant. With the proposed design, there would be 83% power to detect a difference between 50% landing in the control arm and 35% in the intervention arm as significant. A simulation was used to determine the power: 1000 trials were simulated, analyzing each using logistic regression including a fixed effect for the study arm and a random effect for chamber-night. The power was given by the proportion of simulated trials which detected a significant difference between the arms. The analysis assumes eight replicates, 100 mosquitoes released per chamber per night, and a between chamber-night standard deviation of 0.4 on the log odds scale.

### Data analysis

The effect of the intervention was estimated on binary endpoints (landing, KD, mortality at 24 h, and blood feeding) compared to the control arms as odds ratios (OR), presented with 95% confidence intervals. The ORs were estimated using logistic regression analysis including intervention as a fixed effect, and a batch effect (clustering of mosquitoes within the same chamber-night) as a random effect. The 2 × 2 Latin squares were analysed for each intervention separately. Due to the limited number of replicates (eight per arm), covariates for chamber and volunteer were not included but this is not expected to have a substantial impact since the study had a fully balanced design (each treatment occurred an equal number of times in each sequence, and each collector received each treatment an equal number of times). The analysis was conducted in R [47] using the tidyverse packages ‘tidyr’ [48], ‘dplyr’ [49], ‘lme4’ [50], and ‘ggplot2’ [51]. Percent protective efficacy was estimated as $\left(1-OR\right) \times 100.$

## Results

### Insecticide susceptibility testing

AFRIMS’ *An. minimus* (lab strain) and KU’s *An. minimus*’ (CDC laboratory strain) full susceptibility status to deltamethrin 0.05%, etofenprox 0.5%, permethrin 0.75%, and transfluthrin 0.06% were confirmed (100% of mosquitoes tested at both AFRIMS and KU were dead at the 24-h mortality check) [44].

### Quality checks for the SFS- and lab-collected data on intervention impact

The quality of the collected data was verified by comparing replicate data to three replicate criteria for landing, recovery, and mortality rates: 1. above or equal to 95% mosquito recovery in both the control and intervention replicates; 2. less than 10% control mortality at 24 h post-exposure; 3. above or equal to 50% control blood feeding success [44, 45]. Adequate mosquito recovery rates are especially critical to reliably measure secondary outcomes such as KD, post-exposure blood feeding inhibition, and mortality at 24 h. The total number of replicates from both sites that met each replicate criterium improved from round 1 to round 2 (Table 3).

**Table 3:** Percentage of replicates meeting each criterium

Test round	< 10% control mortality at 24-h (%)	> 50% control blood-feeding success (%)	≥ 95% mosquito recovery in control (%)	≥ 95% mosquito recovery intervention (%)
1	78	63	24	10
2	88	100	85	64

### Variability in detected magnitude of effects between research sites and between rounds 1 and 2

The trial results demonstrated variation in the magnitude of effects for several interventions tested and outcomes measured. This was observed between the two research sites, and between R1 and R2. While there were some exceptions, in general, the magnitudes of effects were more pronounced at KU than at AFRIMS, across most interventions, outcome measures, and test rounds (Suppl Figs. 1, 2, 3).

### Product results

In both research sites, all 15 interventions reduced *An. minimus* landing and blood feeding, and induced KD and mortality at 24 h. The combined intervention (BB-new + EtoCL-0-Pi) was the most effective for all measured outcomes. The ORs with 95% confidence intervals and protective efficacies are reported in supplementary tables 1–3.

### Spatial repellents

#### BiteBarrier (BB-new, BB-20, BB-30)

**Fig. 4:**
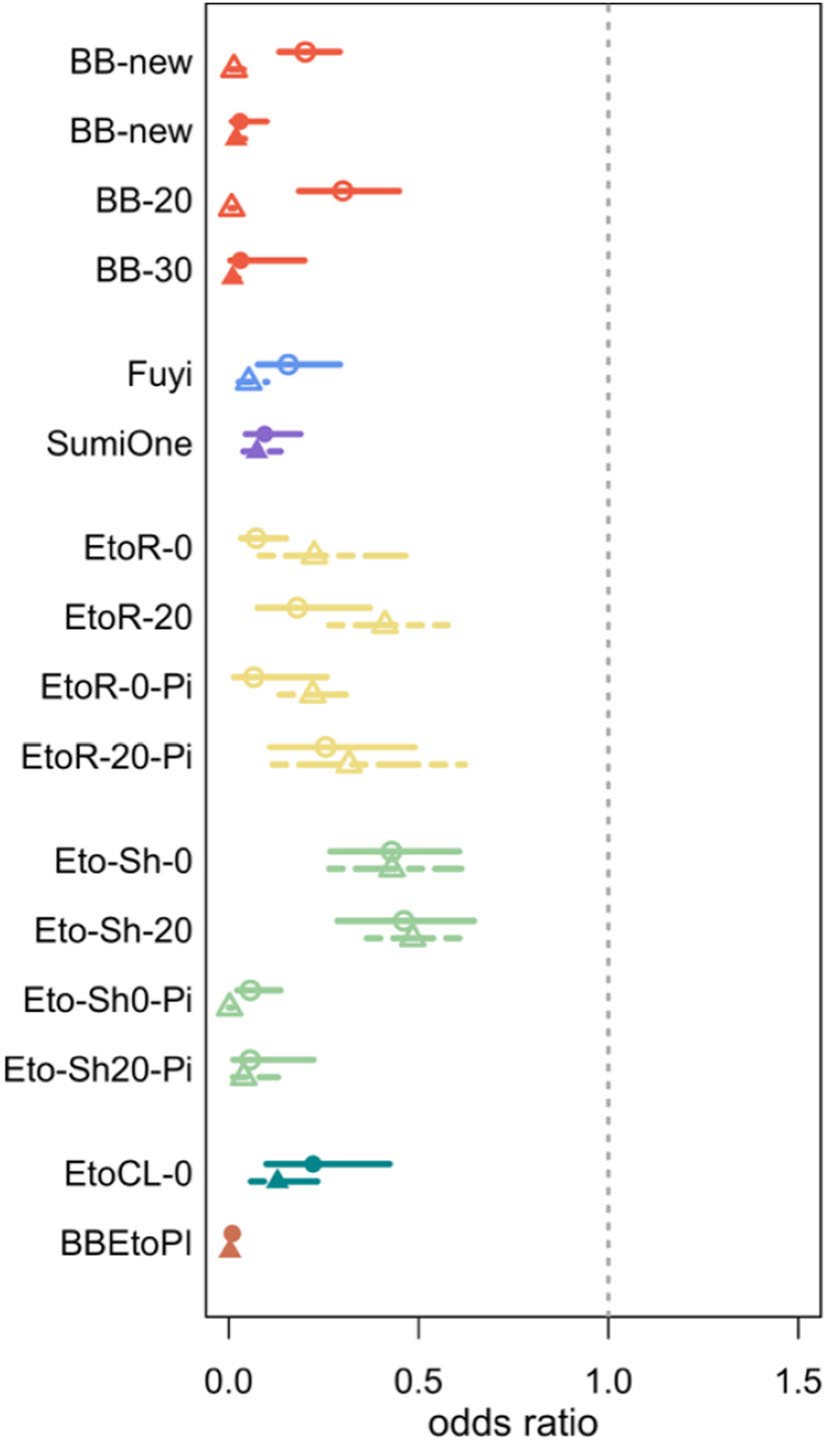
Estimated odds ratios (95% CI) for the effect of each intervention on odds of *Anopheles* landing (AFRIMS represented by circle and solid line; KU represented by triangle and dashed lines)

**Fig. 5:**
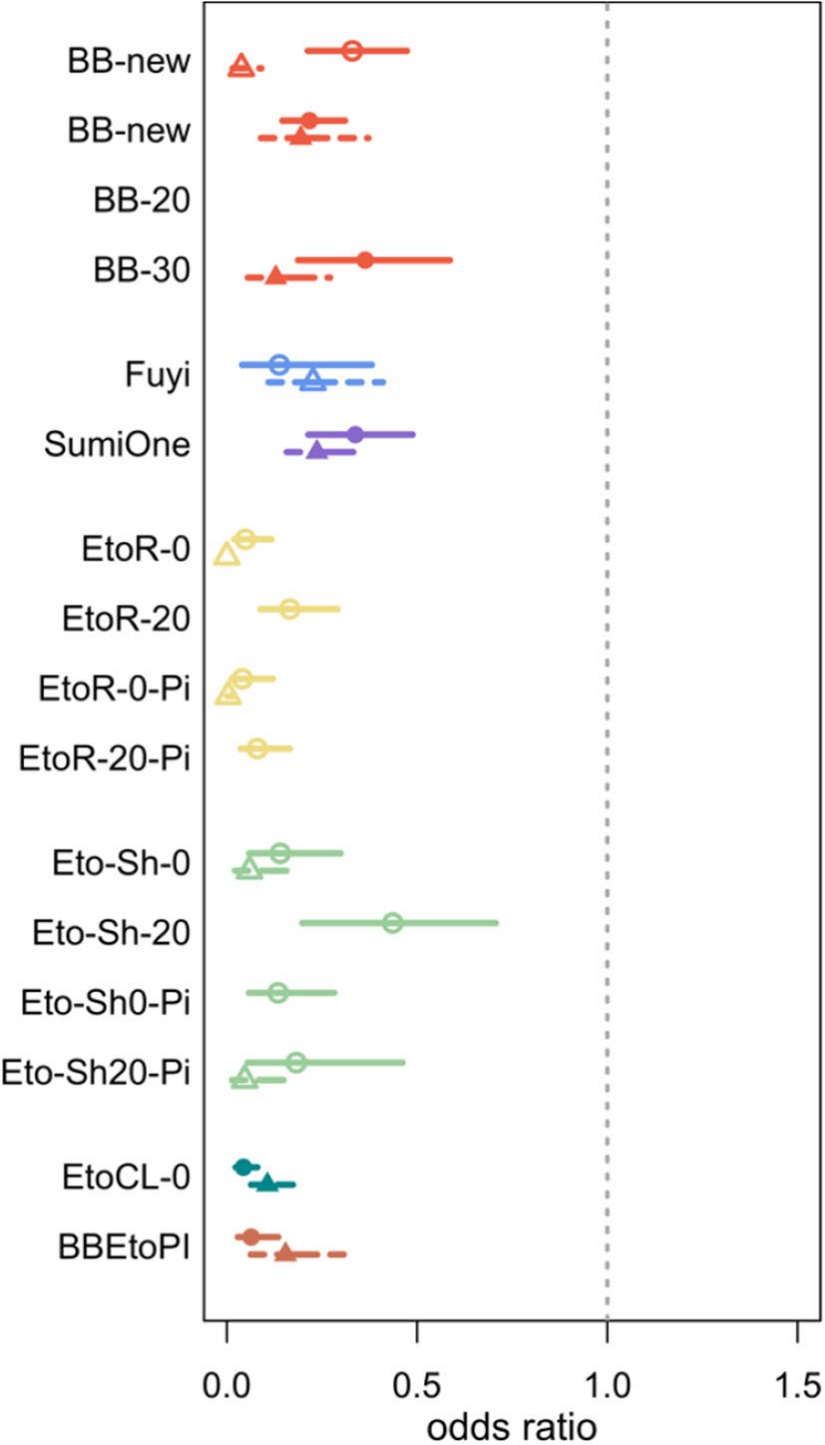
Estimated odds ratios (95% CI) for the effect of each intervention on odds of *Anopheles* immediate, post-exposure blood feeding (AFRIMS represented by circle and solid line; KU represented by triangle and dashed lines)

**Fig. 6:**
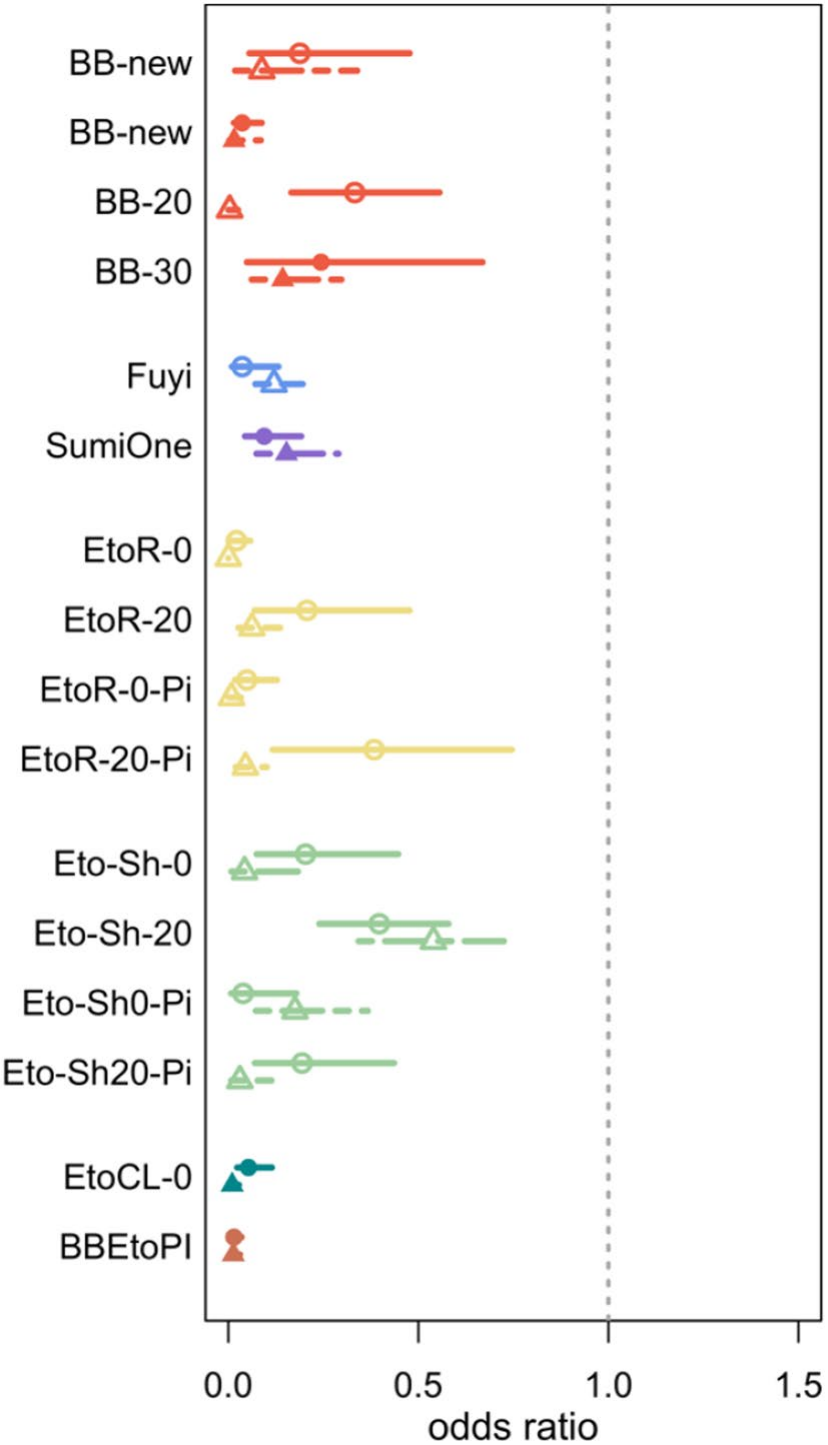
Estimated odds ratios (95% CI) for the effect of each intervention on odds of *Anopheles* survival at 24 h post-exposure (AFRIMS represented by circle and solid line; KU represented by triangle and dashed lines)

**Fig. 7:**
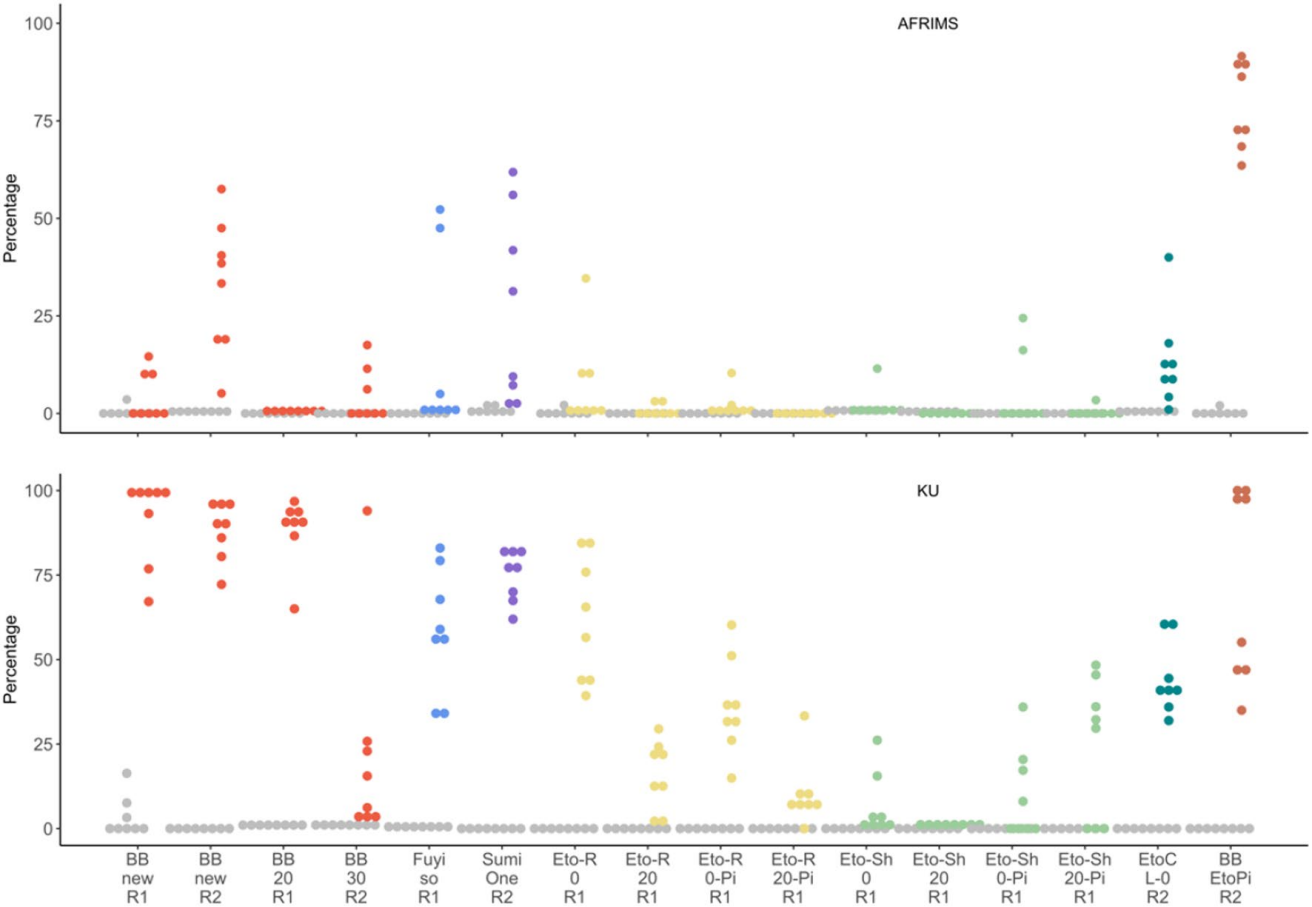
Percentages of *An. minimus* that were knocked down in the intervention (colored) and control arm (grey) replicates at AFRIMS (top) and KU (bottom)

This passive, transfluthrin-based VPSR was effective in reducing *An. minimus* landing and post-exposure blood feeding, as well as inducing KD and mortality at 24 h. The VPSR’s efficacy against landing, blood feeding, survival at 24 h, and in inducing KD was preserved even when aged by 20 and 30 days (blood feeding not measured for BB-20) (Figs. 4, 5, 6, 7; Suppl Tables 1, 2, 3).

#### Fuyi Sin Olor (Fuyi)

This active, transfluthrin-based VPSR aerosol was effective in reducing *An. minimus* landing, blood feeding, and in inducing KD and 24-h mortality, at both KU and AFRIMS (Figs. 4, 5, 6, 7; Suppl Tables 1, 2, 3).

#### SumiOne

At both research sites, this passive, metofluthrin-based VPSR was effective against *An. minimus* landing, blood feeding, and in inducing KD and 24-h mortality (Figs. 4, 5, 6, 7; Suppl Tables 1, 2, 3).

### ITCs

#### Etofenprox-treated ranger uniforms (with or without the 20% picaridin topical repellent)

All etofenprox-treated ranger uniform interventions were effective in reducing landing and blood feeding, and in inducing KD and 24-h mortality. Washed treated ranger uniforms suggested slightly reduced effectiveness against landing (Figs. 4, 5, 6, 7; Suppl Tables 1, 2, 3).

#### Etofenprox-treated civilian clothing (with or without the 20% picaridin topical repellent, long or short trousers)

All etofenprox-treated civilian short clothing interventions and etofenprox-treated civilian long clothing interventions were effective in reducing landing and blood feeding, and in inducing KD and 24-h mortality. Overall, the treated long clothing intervention was more effective than the treated short clothing without topical repellent. However, the unwashed and washed treated short clothing paired with the picaridin had a greater effect against landing than the treated short and long clothing interventions without picaridin (Fig. 4; Suppl Table 1). For the short clothing interventions, there was a decrease in effectiveness against blood feeding and survival at 24 h when washed. In comparison, the washed clothing interventions paired with the picaridin topical repellent indicated increased effectiveness (Figs. 4, 5, 6, 7; Suppl Tables 1, 2, 3).

### *Combined interventions (BB-new* + *EtoCL-0)*

The combined intervention was effective in reducing landing (nearly all risk of landing was removed (Suppl Table 1)) and blood feeding, as well as inducing KD and 24-h mortality (Figs. 4, 5, 6, 7; Suppl Tables 1, 2, 3).

## Discussion

### Intervention modes of action and protective efficacy trends

All interventions substantially reduced *An. minimus* landing and prevented more than 50% of mosquito landing when new or unwashed. In addition to landing reduction, results suggest the potential for all interventions to offer community protection by preventing diversion to nearby unprotected individuals through disarming (knockdown and blood feeding inhibition) of mosquitoes. While the combined intervention had the highest overall protective efficacy, this combined intervention—conceptually designed to be delivered as a forest pack to provide “24-h protection”—is intended to protect real-world users both while inside their homes or temporary shelters and while mobile (outside their homes/shelters). Thus, these interventions may not necessarily all be used simultaneously, but rather as needed, based on individuals’ daily activities.

While the disarming effect was slightly reduced for BB-20 and BB-30 compared to BB-new, the reduction in landing was maintained despite having been aged. In a recent SFS study also evaluating the BiteBarrier, the repellence effect persisted through the testing period of 5 weeks with only a slight decline in efficacy [9]. Understanding the residual efficacy is relevant for informing the timing of product replacement in the field. Subsequent studies are required to assess the protective efficacy of the BiteBarrier beyond the 30-day aging period.

The protective efficacy of etofenprox-treated ranger uniforms did not increase when paired with the 20% picaridin topical repellent, suggesting that the topical repellent is not beneficial for this intervention. Similarly to ITC, topical repellents provide personal protection against mosquito bites through a range of behavioral short-range actions, such as olfactory attractor inhibition [18, 52], and mosquito diversion through non-contact excitorepellency, and/or contact irritancy [20, 21]. The addition of the topical repellent to the treated ranger uniform likely does not enhance the protective efficacy because the long sleeves and long trousers are already providing very high protection against mosquito landing. In contrast, the pairing of picaridin with the etofenprox-treated civilian clothing (short trousers) substantially reduced mosquito landing, indicating a potential benefit of supplementing this intervention with a topical repellent for improved protection.

### Variability in magnitude of effects

This SFS evaluation detected effect signals for all the tested interventions, and the observed patterns of protective efficacy were similar across both research sites. However, the intervention magnitude of effects was generally more pronounced at KU compared to AFRIMS (Figs. 4, 5, 6, 7). Based on the number of sites in this study, it not possible to estimate the variability between sites. Obtaining a precise estimate of intervention effect and its uncertainty would require additional test sites and test replicates. However, the recommended number of test sites and replicates could not be determined based on this SFS evaluation.

The variability in the magnitude of effects in the two research sites could be caused by factors such as weather [9, 36], collector skill, mosquito fitness, and mosquito handling. This SFS evaluation was not designed to assess the effect of such parameters on the estimated intervention effects on landing, KD, blood feeding, and mortality. However, while these SFS evaluations were conducted at both research sites using the same protocol and the same standard operating procedures, it is still plausible that some operational discrepancies between the two research sites remain and account for some of the observed differences in magnitude of effects. Additionally, it is also possible that the observed differences in magnitude of effect are in part due to *Anopheles minimus* strain differences in pyrethroid susceptibility and bionomics profiles. The specific endpoints measured to describe intervention modes of action are affected by mosquito exposure to the active ingredient; as differences in the bionomics of mosquito species/strains affect mosquito-intervention exposure across sites, the magnitude of effects on mosquitoes might also vary by site [42].

Variation in pyrethroid susceptibility across mosquito species and strains is likely to be an important driver of variability in effect size [42, 53, 54]. Some species require higher doses of a given volatile pyrethroid (e.g., metofluthrin, transfluthrin) than others to be repelled [30, 55]. Recent semi-field data indicate that pyrethroid resistance in common African malaria vectors did not prevent volatile transfluthrin at concentrations higher than 5.25 g from reducing landing, although the strain exhibiting the highest phenotypic resistance reduced landing less than the moderately resistant strain [29]. Similar findings were also noted in another semi-field study which examined the efficacy of volatile transfluthrin against a moderately pyrethroid resistant *Anopheles* strain [54], corroborating observations from field studies in Tanzania [7, 8, 56]. Further, species-specific behavioural sensitivity to pyrethroids may occur at concentrations lower than WHO discriminating doses. In fact, the lethal concentrations (LC) that give 99.9% susceptible mosquito mortality (LC_99_) at 24 h post one hour exposure of transfluthrin and metofluthrin are extremely variable between susceptible laboratory strains [57]. In this study, both AFRIMS’ and KU’s *An. minimus* colonies were confirmed susceptible to the WHO discriminating doses of deltamethrin, permethrin, transfluthrin, and permethrin. However, test mosquitoes should also be tested for their susceptibility to pyrethroids at lower doses than the WHO doses because VPSRs exploit lower doses of pyrethroids than ITNs and IRS [55].

Differing levels of anthropophily affect the measured human landing rate [42, 58, 59]. Consequently, repellent interventions might influence the landing of mosquitoes exhibiting higher levels of anthropophily more than mosquitoes that are more opportunistic in their feeding behaviors. Thus, in addition to mosquito pyrethroid susceptibility and sensitivity profiles, it is also relevant to consider the bionomics profiles of the mosquito species and strains targeted, as the modes of action of these interventions are likely species-/strain-dependent, in addition to being dose-dependent [30, 42, 55].

Variability in the magnitude of effects on mosquitoes is observed in ITNs and IRS evaluations in different sites [60, 61]. This was also observed for bite prevention interventions in the present SFS evaluation. At the time of this study, the WHO recommended that testing of novel interventions be conducted at a minimum of two research sites, but this SFS evaluation indicates that more than two research sites are required to better understand intervention modes of actions against *Anopheles* species and strains of various bionomics and resistance profiles. In fact, recently the WHO updated its prequalification (PQ) ITN guidelines and now recommends testing of novel interventions be carried out at three research sites (Module 5, Implementation guidance) [62].

#### Study limitations: possible sources of bias

This SFS evaluation used mosquito landing as a proxy for mosquito biting. Although landing rates are often used as a proxy for biting rates [63], evidence indicates that landing may not systematically equate to biting, as mosquitoes might land but sublethal effects on odour processing inhibit blood feeding [34, 56, 64–66]. In a lab study measuring the landing and biting rates of *Aedes aegypti* against metofluthrin VPSRs, only eight bites for 74 landings were recorded [56]. Another SFS study on transfluthrin’s protective efficacy against *Anopheles gambiae *sensu stricto and *Anopheles funestus* observed higher protection with biting measurements than landing measurements, yet overall protective efficacy, measured by both landing and biting, remained consistent across all species and transfluthrin doses tested [55]. However, ITC primarily works through *contact* irritancy, requiring tarsal contact with the treated surface for mosquitoes to be affected by the active ingredient. In the present SFS evaluation, mosquitoes were offered a blood meal immediately after HLCs to study biting behavior [67] since biting was not permitted at the research sites. Still, it cannot be excluded that measuring only landing rates may lead to an overestimation of ‘biting’, providing an incomplete picture of ITC interventions’ protective effects against mosquito biting. Data from a laboratory study have indicated that permethrin functions by both reducing landing and biting of those mosquitoes that land [68]. Therefore, SFS evaluations in which mosquito biting is also a measured outcome might lead to more accurate measurements of protective efficacy when evaluating interventions that primarily act through contact irritancy (e.g., ITC).

SFS evaluations must control for possible sources of bias, such as weather parameters. For instance, transfluthrin shows a reduction in effectiveness against *Anopheles* landing when temperatures drop below 23 ºC [7, 69]. How the effects differ according to weather conditions could be estimated by conducting SFS experiments at different seasons. This would capture fluctuations in key weather parameters (rainfall, temperature, humidity) that affect both how interventions function and the impact of different environmental conditions on mosquito behaviour [9].

Other possible sources of bias in this study include low mosquito recapture in round 1, as elevated numbers of test mosquitoes that are unaccounted for can lead to inaccurate and imprecise outcome measurements, especially evaluation of mosquito knockdown and mortality. Therefore, it is critical to ensure high recapture in tandem with elevated mosquito avidity [9].

### The role of SFS evaluations for product testing

SFS intervention evaluations enable a baseline understanding of how interventions affect mosquitoes, and help prioritize which interventions merit further investigation [70]. Following SFS evaluations, interventions must be tested in the field in contrasting geographical field settings characterized by varying mosquito bionomics and susceptibility profiles. Subsequent to the present SFS evaluation, Vajda et al. [40] tested a subset of these interventions against wild *Anopheles* landing in a controlled field setting in Mondulkiri Province, Cambodia. The field results supported the SFS results on landing inhibition: the passive, transfluthrin-based VPSR (BiteBarrier) and the combined BiteBarrier + treated clothing interventions provided the most protection against mosquito landing, followed by the treated clothing with topical repellent interventions [40].

SFS evaluations are a critical first step towards understanding how reductions in mosquito landings can translate into reduced malaria burden, because they help describe their impact on vectoral capacity through the collection of endpoints beyond landing. The findings of this SFS evaluation are encouraging. In a modeling assessment using results from this SFS evaluation, Fairbanks et al. [41] extended the Denz et al*.* [26] framework by using the mosquitoes that had not been collected by HLC, and which were aspirated from the walls and floor of the SFS chambers and including post-exposure blood feeding to parameterize disarming and preprandial mortality enabling the assessment of both personal and community protections. The model indicated that these interventions substantially reduced vectoral capacity by reducing landing, blood feeding and killing a proportion of mosquitoes. The model also demonstrated that blood feeding measurements are essential for better estimating community protection, because measuring blood feeding clarifies whether mosquitoes not captured via HLCs were repelled and continued host-seeking elsewhere, or if they were disarmed [41]. These modeling evaluations corroborate findings from other recent SFS experiments [7–9, 23, 27, 28] and field studies of VPSRs [30, 37, 71]. Given these recent studies, spatial repellents are increasingly recognized as having high potential for public health use, but further evidence of epidemiological impact is needed for the WHO to establish a policy recommendation for these insecticide-based interventions [72].

## Conclusion

The SFS evaluation indicates the effectiveness of VPSR and ITC interventions in reducing *An. minimus* landings. This aligns with the related field evaluation in Cambodia of a subset of these interventions. Furthermore, this SFS evaluation highlights the potential of VPSRs and ITCs to offer community protection by disarming mosquitoes, thus protecting both users and nearby non-users. Continued investigation into the residual efficacy of these interventions is required. Variability in the magnitude of effects across research sites was observed and was possibly influenced by both operational discrepancies and *An. minimus* strain differences in bionomic and pyrethroid susceptibility profiles. Despite study limitations, such as using landing as a proxy for biting, the findings together with model predictions underscore the potential of these interventions—which were traditionally only considered to provide personal protection—to reduce malaria burden among users and non-users if applied at scale. SFS evaluations are an imperative initial step in the product evaluation pathway to understand their modes of action, guiding further field testing and providing valuable insights into the mode of action of interventions and their potential impact on vectoral capacity. While additional evidence of epidemiological impact is required for the WHO to formulate public health policy recommendations for these interventions, the encouraging results from this evaluation, backed by subsequent field tests, suggest a promising avenue for reducing malaria incidence.

## Supplementary Information

Additional file 1

## Data Availability

Correspondence and requests for data should be directed to EAV.
