# Supplementary material for: The effect of novel mosquito bite prevention tools on Anopheles minimus landing and key secondary endpoints: semi-field evaluations in Thailand
Source: Malar J. 2024 Dec 18;23:387. doi: 10.1186/s12936-024-05188-3 (PMC11656824; doi:10.1186/s12936-024-05188-3)
Supplement: Supplementary file 1 — Additional file 1 [file 12936_2024_5188_MOESM1_ESM.docx]

**Supplementary Materials**

**Table S1** Measured **landing** estimated ORs and protected efficacies (presented with 95% confidence intervals) for each intervention

| Intervention (Test round) | AFRIMS | | | KU | | |
| --- | --- | --- | --- | --- | --- | --- |
|  | **OR** | **p-value** | **% Protective efficacy** | **OR** | **p-value** | **% Protective efficacy** |
| BiteBarrier (new) (BB-new) (R1) | 0.20 (0.13 – 0.29) | <0.001 | 80 (71 – 87) | 0.01 (0.00 – 0.05) | <0.001 | 99 (95 – 100) |
| BiteBarrier (new) (BB-new) (R2) | 0.03 (0.01 – 0.10) | <0.001 | 97 (90 – 99) | 0.02 (0.01 – 0.05) | <0.001 | 98 (95 – 99) |
| BiteBarrier (aged 20 days) (BB-20) (R1) | 0.30 (0.19 – 0.45) | 0.009 | 70 (55 – 81) | 0.01 (0.00 – 0.01) | <0.001 | 99 (99 – 100) |
| BiteBarrier (aged 30 days) (BB-30) (R2) | 0.03 (0.00 – 0.20) | 0.001 | 97 (80 – 100) | 0.01 (0.00 – 0.03) | <0.001 | 99 (97 – 100) |
| Fuyi Sin Olor (Fuyi) (R1) | 0.16 (0.08 – 0.29) | <0.001 | 84 (71 – 92) | 0.05 (0.03 – 0.10) | <0.001 | 95 (90 – 97) |
| SumiOne (new) (R2) | 0.09 (0.04 – 0.19) | <0.001 | 91 (81 – 96) | 0.07 (0.04 – 0.14) | <0.001 | 93 (86 – 96) |
| Etofenprox-treated ranger uniform (0 washes)  (EtoR-0) (R1) | 0.07 (0.03 – 0.15) | <0.001 | 93 (85 – 97) | 0.23 (0.08 – 0.49) | 0.042 | 77 (51 – 92) |
| Etofenprox-treated ranger uniform (20 washes)  (EtoR-20) (R1) | 0.18 (0.08 – 0.37) | 0.003 | 82 (63 – 92) | 0.41 (0.26 – 0.58) | 0.3 | 59 (42 – 74) |
| Etofenprox-treated ranger uniform with 20% picaridin (0 washes) (EtoR-0-Pi) (R1) | 0.07 (0.01 – 0.26) | 0.001 | 93 (74 – 99) | 0.22 (0.13 – 0.34) | <0.001 | 78 (66 – 87) |
| Etofenprox-treated ranger uniform with 20% picaridin (20 washes) (EtoR-20-Pi) (R1) | 0.26 (0.11 – 0.49) | 0.041 | 74 (51 – 89) | 0.32 (0.12 – 0.62) | 0.235 | 68 (38 – 88) |
| Etofenprox-treated civilian clothing (short-sleeved shirts and short trousers) (0 washes) (EtoC-0) (R1) | 0.43 (0.27 – 0.61) | 0.436 | 57 (39 – 73) | 0.43 (0.26 – 0.61) | 0.455 | 57 (39 – 74) |
| Etofenprox-treated civilian clothing (short-sleeved shirts and short trousers) (20 washes) (EtoC-20) (R1) | 0.46 (0.29 – 0.65) | 0.686 | 54 (35 – 71) | 0.48 (0.36 – 0.61) | 0.808 | 52 (39 – 64) |
| Etofenprox-treated civilian clothing (short-sleeved shirts and short trousers (knee-length)) with 20% picaridin (0 washes) (EtoC-0-Pi) (R1) | 0.06 (0.02 – 0.14) | <0.001 | 94 (86 – 98) | 0.00 (0.00 – 0.01) | <0.001 | 100 (99 – 100) |
| Etofenprox-treated civilian clothing (short-sleeved shirts and short trousers (knee-length)) with 20% picaridin (20 washes) (EtoC-20-Pi) (R1) | 0.06 (0.01 – 0.22) | <0.001 | 94 (78 – 99) | 0.04 (0.01 – 0.13) | <0.001 | 96 (87 – 99) |
| Etofenprox-treated civilian clothing (short-sleeved shirts and long trousers) with 20% picaridin (0 washes)  (EtoCL-0-Pi) (R2) | 0.22 (0.10 – 0.42) | 0.009 | 78 (58 – 90) | 0.13 (0.06 – 0.26) | <0.001 | 87 (74 – 94) |
| Combined interventions (VPSR1-new + EtoCL-0-Pi) (R2) | 0.01 (0.00 – 0.02) | <0.001 | 99 (98 – 100) | 0.00 (0.00 – 0.01) | <0.001 | 100 (99 – 100) |

**Table S2** Measured **blood feeding** ORs and associated protected efficacies (presented with 95% confidence intervals) for each intervention

| Intervention (Test round) | AFRIMS | | | KU | | |
| --- | --- | --- | --- | --- | --- | --- |
|  | **OR** | **p-value** | **% Protective efficacy** | **OR** | **p-value** | **% Protective efficacy** |
| BiteBarrier (new) (BB-new) (R1) | 0.33 (0.21 – 0.47) | 0.021 | 67 (53 – 79) | 0.04 (0.02 – 0.09) | <0.001 | 96 (91 – 98) |
| BiteBarrier (new) (BB-new) (R2) | 0.22 (0.15 – 0.31) | <0.001 | 78 (69 – 85) | 0.19 (0.09 – 0.37) | 0.002 | 81 (63 – 91) |
| BiteBarrier (aged 20 days) (BB-20) (R1) | Not measured | - | - | Not measured | - | - |
| BiteBarrier (aged 30 days) (BB-30) (R2) | 0.36 (0.19 – 0.59) | 0.227 | 64 (41 – 81) | 0.13 (0.06 – 0.27) | <0.001 | 87 (73 – 94) |
| Fuyi Sin Olor (Fuyi) (R1) | 0.14 (0.04 – 0.38) | 0.008 | 86 (62 – 96) | 0.23 (0.11 – 0.41) | 0.005 | 77 (59 – 89) |
| SumiOne (new) (R2) | 0.34 (0.21 – 0.49) | 0.035 | 66 (51 – 79) | 0.24 (0.16 – 0.34) | <0.001 | 76 (66 - 840 |
| Etofenprox-treated ranger uniform (0 washes)  (EtoR-0) (R1) | 0.05 (0.02 – 0.12) | <0.001 | 95 (88 – 98) | 0.00* |  | 100* |
| Etofenprox-treated ranger uniform (20 washes)  (EtoR-20) (R1) | 0.17 (0.09 – 0.29) | <0.001 | 96 (88 – 99) | Not measured | - | - |
| Etofenprox-treated ranger uniform with 20% picaridin (0 washes) (EtoR-0-Pi) (R1) | 0.04 (0.01 – 0.12) | <0.001 | 96 (88 – 99) | 0.00 (0.00 – 0.02) | <0.001 | 100 (98 – 100) |
| Etofenprox-treated ranger uniform with 20% picaridin (20 washes) (EtoR-20-Pi) (R1) | 0.08 (0.04 – 0.17) | <0.001 | 92 (83 – 96) | Not measured | - | - |
| Etofenprox-treated civilian clothing (short-sleeved shirts and short trousers) (0 washes) (EtoC-0) (R1) | 0.14 (0.06 – 0.30) | <0.001 | 86 (70 – 94) | 0.06 (0.02 – 0.16) | <0.001 | 94 (84 – 98) |
| Etofenprox-treated civilian clothing (short-sleeved shirts and short trousers) (20 washes) (EtoC-20) (R1) | 0.44 (0.20 – 0.71) | 0.657 | 56 (29 – 80) | Not measured | - | - |
| Etofenprox-treated civilian clothing (short-sleeved shirts and short trousers (knee-length)) with 20% picaridin (0 washes) (EtoC-0-Pi) (R1) | 0.13 (0.06 – 0.28) | <0.001 | 87 (72 – 94) | Not measured | - | - |
| Etofenprox-treated civilian clothing (short-sleeved shirts and short trousers (knee-length)) with 20% picaridin (20 washes) (EtoC-20-Pi) (R1) | 0.18 (0.05 – 0.46) | 0.029 | 82 (54 – 95) | 0.05 (0.01 – 0.15) | <0.001 | 95 (85 – 99) |
| Etofenprox-treated civilian clothing (short-sleeved shirts and long trousers) with 20% picaridin (0 washes)  (EtoCL-0-Pi) (R2) | 0.04 (0.02 – 0.08) | <0.001 | 96 (92 – 98) | 0.11 (0.06 – 0.17) | <0.001 | 89 (83 – 94) |
| Combined interventions (VPSR1-new + EtoCL-0-Pi) (R2) | 0.06 (0.03 – 0.14) | <0.001 | 94 (86 – 97) | 0.15 (0.06 – 0.33) | 0.001 | 85 (67 – 94) |

*****CI could not be estimated due to the data being 0

**Table S3** Measured **24-hour mortality** ORs and associated protected efficacies (presented with 95% confidence intervals) for each intervention

| Intervention (Test round) | AFRIMS | | | KU | | |
| --- | --- | --- | --- | --- | --- | --- |
|  | **OR** | **p-value** | **% Protective efficacy** | **OR** | **p-value** | **% Protective efficacy** |
| BiteBarrier (new) (BB-new) (R1) | 0.19 (0.06 – 0.48) | 0.04 | 81 (52 – 94) | 0.09 (0.02 – 0.34) | 0.006 | 91 (66 – 98) |
| BiteBarrier (new) (BB-new) (R2) | 0.04 (0.01 – 0.09) | <0.001 | 96 (91 – 99) | 0.02 (0.00 – 0.09) | <0.001 | 98 (91 – 100) |
| BiteBarrier (aged 20 days) (BB-20) (R1) | 0.56 (0.17 – 0.33) | 0.137 | 67 (44 – 83) | 0.00 (0.00 – 0.03) | <0.001 | 100 (97 – 100) |
| BiteBarrier (aged 30 days) (BB-30) (R2) | 0.24 (0.05 – 0.67) | 0.226 | 76 (33 – 95) | 0.14 (0.06 – 0.30) | <0.001 | 86 (70 – 94) |
| Fuyi Sin Olor (Fuyi) (R1) | 0.04 (0.01 – 0.13) | <0.001 | 96 (87 – 99) | 0.12 (0.07 – 0.20) | <0.001 | 88 (80 – 93) |
| SumiOne (new) (R2) | 0.09 (0.04 – 0.19) | <0.001 | 91 (81 – 96) | 0.15 (0.07 – 0.29) | <0.001 | 85 (71 – 93) |
| Etofenprox-treated ranger uniform (0 washes)  (EtoR-0) (R1) | 0.02 (0.01 – 0.06) | <0.001 | 98 (94 – 99) | 0.00 (0.00 – 0.00) | <0.001 | 100 (100 – 100) |
| Etofenprox-treated ranger uniform (20 washes)  (EtoR-20) (R1) | 0.21 (0.07 – 0.48) | 0.035 | 79 (52 – 93) | 0.06 (0.03 – 0.14) | <0.001 | 94 (86 – 97) |
| Etofenprox-treated ranger uniform with 20% picaridin (0 washes) (EtoR-0-Pi) (R1) | 0.05 (0.02 – 0.13) | <0.001 | 95 (87 – 98) | 0.01 (0.00 – 0.03) | <0.001 | 99 (97 – 100) |
| Etofenprox-treated ranger uniform with 20% picaridin (20 washes) (EtoR-20-Pi) (R1) | 0.38 (0.12 – 0.75) | 0.549 | 62 (25 – 88) | 0.05 (0.02 – 0.10) | <0.001 | 95 (90 – 98) |
| Etofenprox-treated civilian clothing (short-sleeved shirts and short trousers) (0 washes) (EtoC-0) (R1) | 0.20 (0.07 – 0.45) | 0.020 | 80 (55 – 93) | 0.04 (0.01 – 0.19) | <0.001 | 96 (81 – 99) |
| Etofenprox-treated civilian clothing (short-sleeved shirts and short trousers) (20 washes) (EtoC-20) (R1) | 0.40 (0.24 – 0.58) | 0.266 | 60 (42 – 76) | 0.54 (0.34 – 0.72) | 0.701 | 46 (28 – 66) |
| Etofenprox-treated civilian clothing (short-sleeved shirts and short trousers (knee-length)) with 20% picaridin (0 washes) (EtoC-0-Pi) (R1) | 0.04 (0.01 – 0.18) | <0.001 | 96 (82 – 99) | 0.18 (0.07 – 0.37) | 0.002 | 82 (63 – 93) |
| Etofenprox-treated civilian clothing (short-sleeved shirts and short trousers (knee-length)) with 20% picaridin (20 washes) (EtoC-20-Pi) (R1) | 0.19 (0.07 – 0.44) | 0.017 | 81 (56 – 93) | 0.03 (0.01 – 0.11) | <0.001 | 97 (89 – 99) |
| Etofenprox-treated civilian clothing (short-sleeved shirts and long trousers) with 20% picaridin (0 washes)  (EtoCL-0-Pi) (R2) | 0.05 (0.02 – 0.11) | <0.001 | 95 (89 – 98) | 0.01 (0.00 – 0.03) | <0.001 | 99 (97 – 100) |
| Combined interventions (VPSR1-new + EtoCL-0-Pi) (R2) | 0.01 (0.01 – 0.03) | <0.001 | 99 (97 – 100) | 0.01 (0.01 – 0.03) | <0.001 | 99 (97 – 100) |

| 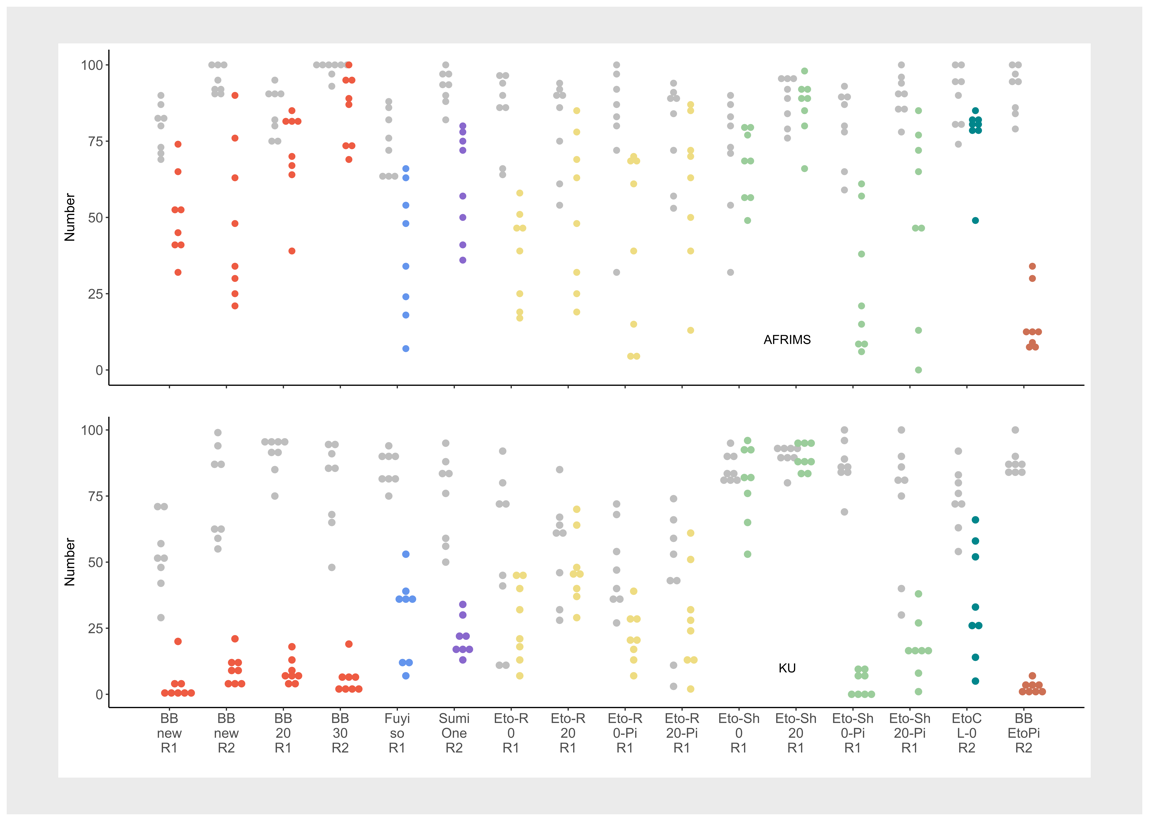  **Fig S1** Number of mosquitoes (out of 100 mosquitoes released per arm) captured via HLCs in the interventions (colored) and their controls (grey) at AFRIMS (top) and KU (bottom) | 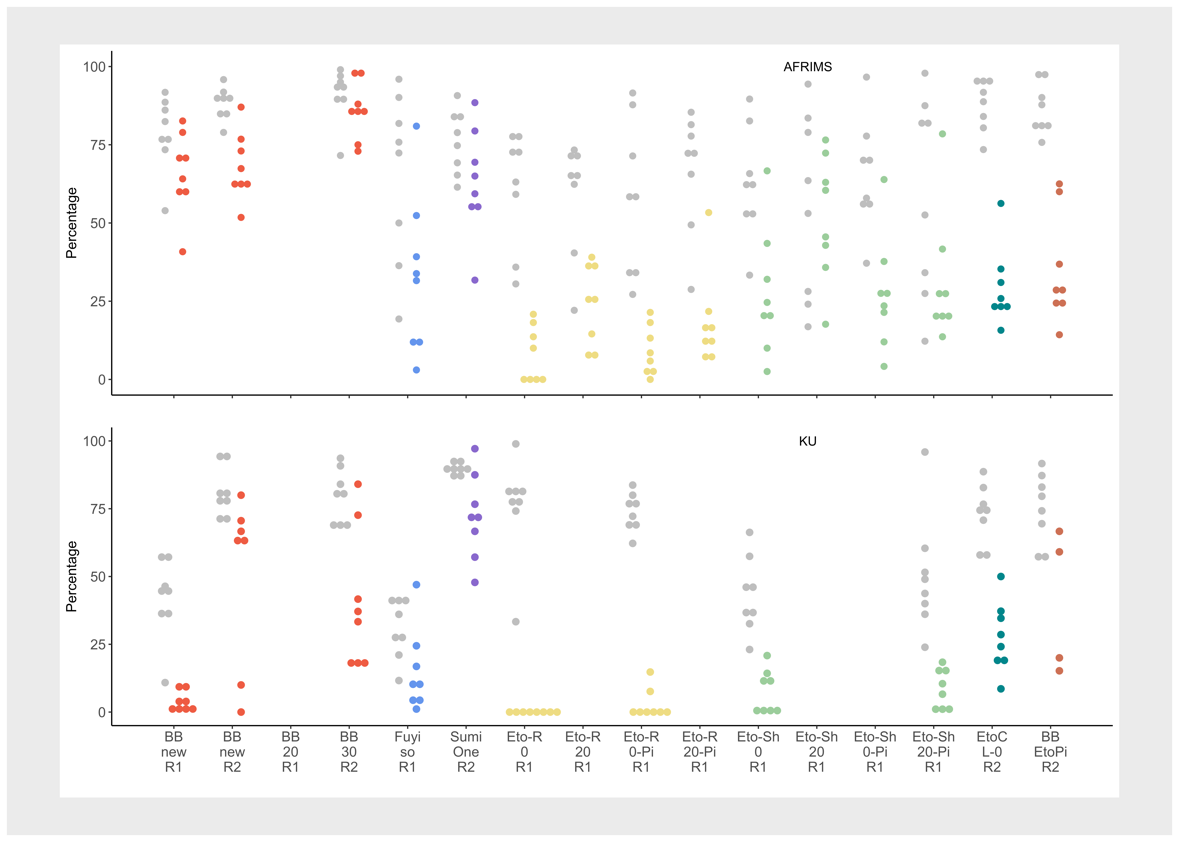  **Fig S2** Proportions of mosquitoes that blood fed post-exposure in the interventions  (colored) and their controls (grey) at AFRIMS (top) and KU (bottom). |
| --- | --- |

| 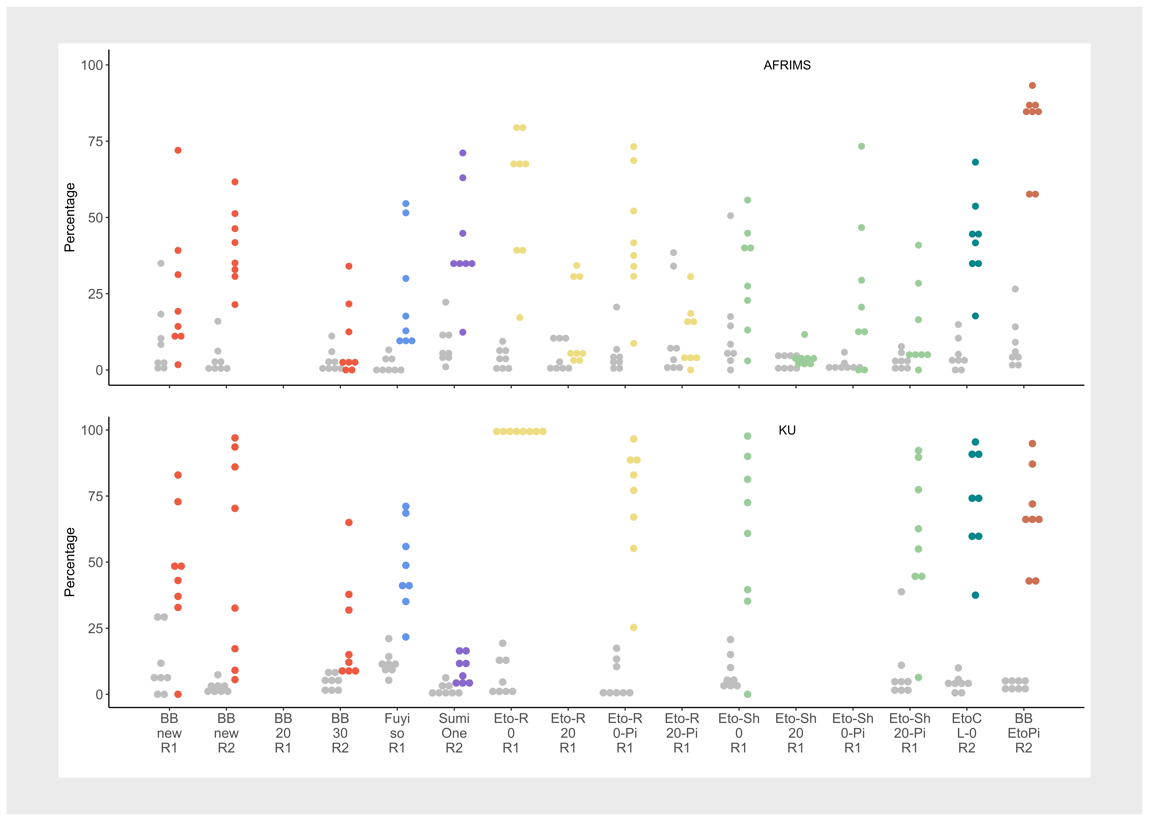  **Fig S3** Proportions of mosquitoes that were dead a 24 hours post-exposure in the  interventions (colored) and their controls (grey) at AFRIMS (top) and KU (bottom). |  |
| --- | --- |
